# First Report of IgG4 Related Disease Primary Presenting as Vertebral Bone Marrow Lesions

**DOI:** 10.3389/fimmu.2019.01910

**Published:** 2019-08-13

**Authors:** Debby van den Elshout-den Uyl, Clothaire P. E. Spoto, Mirthe de Boer, Tim Leiner, Helen L. Leavis, Roos J. Leguit

**Affiliations:** ^1^Department of Rheumatology and Clinical Immunology, University Medical Center Utrecht, Utrecht, Netherlands; ^2^Department of Pathology, University Medical Center Utrecht, Utrecht, Netherlands; ^3^Department of Radiology, University Medical Center Utrecht, Utrecht, Netherlands

**Keywords:** IgG4-related disease, spine, MRI, bone marrow, vertebra, plasma cell, histology, immunhistochemistry

## Abstract

IgG4-related disease is a fibro-inflammatory disorder characterized by swelling of tissues and affected organs accompanied by the development of scar tissue (fibrosis) and infiltration by IgG4 positive plasma cells. Almost any organ can be affected, including, but rarely, bone marrowinvolvement. Here we present a case of a 76-year-old male with IgG4-related disease presenting primarily with vertebral bone marrow lesions. Histopathology showed the typical features of storiform fibrosis, and increased IgG4 positive plasma cells. Treatment with corticosteroids significantly improved wellbeing and resolved lesion size on MRI.

## Introduction

Immunoglobulin G4-related disease (IgG4-RD) is a relatively new fibro-inflammatory condition characterized by tumefactive lesions, a dense lymphoplasmacytic infiltrate rich in IgG4 positive plasma cells, storiform fibrosis, and often elevated IgG4 serum levels. It was first reported in the pancreas and over the years, this entity was recognized in a variety of other organs, with pancreas, bile ducts, salivary and lacrimal glands and lungs being the most common sites (1–3). We here describe a rare case of IgG4-RD presenting primarily in the bone marrow of the vertebrae. The diagnosis was based on histopathology and supported by clinical and laboratory findings.

A 76 year old male was referred to our clinic for a diagnostic second opinion with complaints of fatigue, increased exhaustion after physical exercise, weight loss, and night sweats. Physical examination, including neurological examinations, revealed no abnormalities. Laboratory testing showed an increased erythrocyte sedimentation rate (ESR) of 78 mm/h (normal range 1–11 mm/h) and C-reactive protein (CRP) of 77 mg/L (normal range 0–10 mg/L). Further evaluation demonstrated a polyclonal hypergammaglobulinemia with increased serum kappa and lambda light chains and negative M-protein. The chest X-ray was normal but magnetic resonance imaging (MRI) showed multiple hypodense bone lesions in the thoracic and lumbar spine and in the iliac crest. A bone marrow biopsy of the iliac crest, including immunophenotyping, was negative for malignancy. A first biopsy of a thoracic vertebral body was performed showing normal marrow with a focus of fibrosis with a slight increase in polytypic plasma cells, eosinophils and mast cells with a normal morphology. In this fibrotic area there was no pre-existing hematopoiesis. Elaborate immunohistochemical analysis excluded a hematologic malignancy, metastatic carcinoma and systemic mastocytosis. At that time IgG4 staining was not performed.

**Figure 1:**
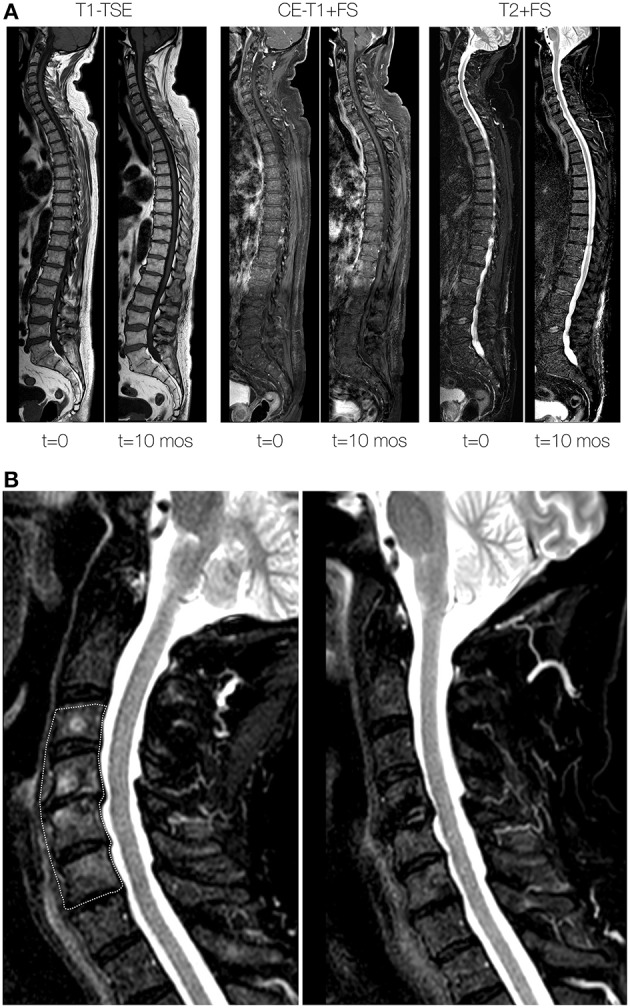
Vertebral lesions at baseline and during follow-up. **(A)** Total spine sagittal reconstructions of T1-weighted turbo spin-echo (TSE; left 2 panels), contrast-enhanced water-selective T1-weighted TSE (middle two panels) and T2-weighted sequences (right two panel) at baseline (t = 0) and 10 months (t = 10 mos) after initiation of treatment. At baseline, note the mottled appearance and lower signal intensity of the vertebral bodies on the T1-weighted sequences at baseline. At 10 months after treatment the signal intensity has returned to near normal signal intensity. **(B)** Zoomed detail of T2-weighted sequences at baseline (left) and 10 months after treatment (right) of the cervical spine. The area outlined by the dashed area at baseline signifies abnormally high signal intensity of the vertebral bone marrow, which returned to normal values at 10 months after treatment.

**Figure 2:**
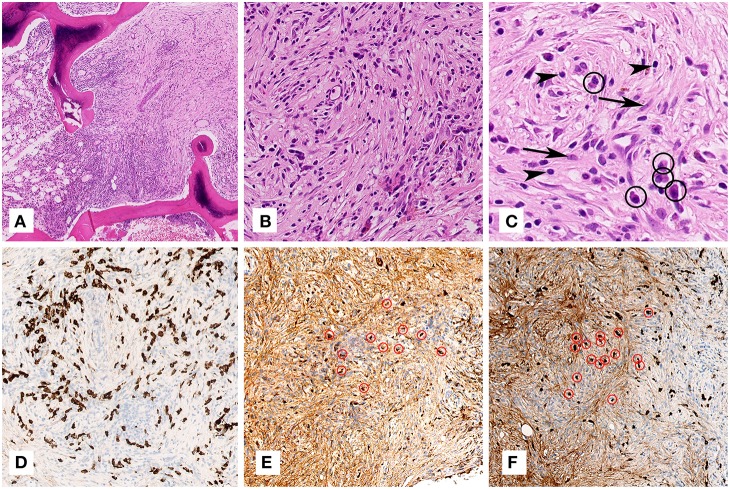
Microscopic examination of the lumbar vertebra (biopsy). **(A)** On overview, the marrow in between the bony trabeculae shows a quite sharp demarcation between normal bone marrow (one third on the left) and the fibrotic lesion (two thirds on the right) (H&E staining, 79x magnification). **(B)** Higher magnification of the lesion shows fibrosis rich in fibroblasts and arranged in a somewhat storiform fashion with admixed plasma cells and lymphocytes (H&E staining, 400x magnification). **(C)** Highest magnification shows several fibroblast (arrows), plasma cells (encircled) and lymphocytes (arrow heads) (H&E staining, 800x magnification). **(D)** Immunohistochemistry for CD138 shows in dark brown the numerous plasma cells (CD138 immunohistochemical staining, 400x magnification). **(E,F)** Immunohistochemistry for IgG **(E)** and IgG4 **(F)** shows in dark brown numerous IgG and IgG4 positive plasma cells, respectively, some of which encircled in red (IgG and IgG4 immunohistochemical stainings, 200x magnification).

During the following months, the constitutional symptoms, and the elevated inflammation markers persisted, and a mild normocytic anemia and leucocytosis developed (Figure 1). A subsequent PET-CT scan showed multiple and diffuse FDG-avid bone lesions along the spinal cord that were suspect for metastasis and a new epidural mass developed in the thoracic spine extending from the 10th to the 11th thoracic vertebra without compression of the spinal cord. On computed tomography (CT) imaging, the lesions appeared sclerotic. Again, a biopsy was performed, this time of a lumbar vertebral lesion (Figure 2). A quite sharp demarcation was seen between normal bone marrow and the fibrotic lesion (Figure 2A). The fibrosis in this biopsy was comparable to the previous one, but appeared to be more storiform (Figure 2B). The fibrotic focus had a relatively high cellularity, containing fibroblasts, plasma cells and lymphocytes (Figure 2C). Occasional macrophages were seen but eosinophils were rare. Immunohistochemical analysis demonstrated <10% plasma cells in pre-existing bone marrow, but around 25% plasma cells were present in the fibrotic lesion (Figure 2D). The plasma cells showed polyclonal kappa and lambda light chain expression. Many of the plasma cells were IgG and IgG4 positive (Figures 2E,F, respectively). Per high power field (HPF), 86 IgG4 positive plasma cells were present on a total of 111 IgG positive plasma cells (ratio 0.78). The following immunohistochemical stains were used to rule other lesions and malignancy: CKAE1/3 (carcinoma), S100 (melanoma and neuronal malignancies), CD1a and langerine (Langerhanscel histiocytosis), CD117 (mastocytosis), PAX5, CD3, and CD30 (B- and T-cell lymphoma), CD21 and CD23 (follicular dendritic cell sarcoma) and CD34 (several sarcomas, solitary fibrous tumor). IgG4–RD was considered and serum levels of IgG4 at this point were found elevated (e.g., 120 mg/dL).His IgG total level was 104, IgG1 49, IgG2 459, IgG3 46 mg/dL (all within normal range). Thereupon treatment with glucocorticosteroids (prednisolone starting dose 40 mg/day) was initiated, with subsequent disappearance of symptoms and normalization of weight, serum hemoglobin and ESR. The vertebral lesions and epidural mass decreased in size over time (Figure 1) and IgG4 level turned to normal (58 mg/dL) with the patient being in persistent remission on low dose prednisolone.

## Discussion

IgG4-RD is fibro-inflammatory condition more prevalent in middle-aged and elderly males. The major symptoms of IgG4-RD are related to the mass effect and thus commonly present with site specific symptoms, often raising suspicion of malignancy. Site specific symptoms include, but are not limited to: sicca features, respiratory symptoms, pruritus, eczema, abdominal pain, and diarrhea. 10–20% of patients have a history of atopy (4) A small number of IgG4-RD patients experience constitutional symptoms such as fever and weight loss, and in some asymptomatic patients a mass lesion is only found incidentally on imaging. Typically, ESR and CRP are not elevated. Elevated IgG4 serum levels are often reported, but 30–80% of biopsy proven IgG4-RD had normal serum levels (3, 5, 6).

Involvement of the central nervous system in IgG4-RD is rare, and often manifests as hypertrophic pachymeningitis, in which there is a focally or diffusely thickened dura mater. In this disease, the cranial part of the dura is predominantly involved, but over the past years several case reports have been published describing involvement of the spinal dura (2, 5–10). Most of these patients with hypertrophic pachymeningitis had back pain and neurological complaints (5–7, 11).

Bone marrow involvement has previously been described in patients with IgG4-RD as FDG-PET/CT bone marrow FDG-avidity but without lytic or sclerotic bone regions on plain CT (12) and/or as plasmacytosis on histopathological examination with increased IgG4 positive plasma cells with otherwise unremarkable bone marrow without fibrosis (12–14). Furthermore, IgG4-RD lesions have been described as a costochondral mass (15), both with the typical features of storiform fibrosis and increased IgG4 positive plasma cells.

Our case-report describes a patient with sclerotic, PET-positive IgG4-RD lesions in the vertebrae as primary manifestation of the disease. The clinical differential diagnosis of lesions in the vertebral body is broad, ranging from benign diagnoses (e.g., haemangioma, Paget's disease, osteonecrosis), to malignancies (e.g., metastases, multiple myeloma, lymphoma, sarcoma), and infectious causes (e.g., osteomyelitis, mycobacterial infection). In our case, elaborate diagnostic work-up, including laboratory, and radiological results and histopathological findings, did not reveal a malignancy. Based on the findings of the second biopsy, IgG4-RD was diagnosed. Histologically, mastocytosis was excluded based on the absence of aggregates or spindled mast cells. Beside, mast cells in the fibrotic focus had no aberrant immunohistochemical profile (CD2 and CD25 were negative) and no KIT mutation was detected using Next Generation Sequencing. Plasma cell dyscrasia was ruled out based on the polytypic immunohistochemical expression of kappa and lambda light chain and the absence of a B-cell clone on clonality analysis.

In general, the diagnosis of IgG4-RD is made based on specific histopathological features, with the presence of at least 2 of the following 3 major criteria: (1) dense lymphoplasmacytic infiltrate, (2) fibrosis with a focal or diffuse storiform pattern, and (3) obliterative phlebitis. In addition, an increased IgG4 positive plasma cell / IgG4 positive plasma cell ratio of at least 0.4 in the affected tissue should be present (8, 16). An absolute quantification of total IgG4 positive plasma cells per HPF is also advised, but the cutoff varies based on the localization of the disease. Cutoffs varying between >10 to >200 IgG4 positive plasma cells / HPF have been proposed, which makes this a tricky criterion to use in daily practice. It is important to note that, without being a diagnostic criterion for IgG4-RD, the bulk of the inflammatory infiltrate usually consists of T lymphocytes, predominantly of the CD4+ lineage. A less conspicuous component of B lymphocytes (with or without the formation of lymphoid follicles) with intermixed macrophages can also be present. Eosinophils are prominent and are almost invariably present. Necrosis, granulomas and multinucleated giant cells and necrosis however, are absent, and their presence should trigger the pathologist to consider an alternative diagnosis. The same applies to the presence of neutrophils, which are usually not found, with the exception of intra-alveolar neutrophils in IgG4-RD of the lungs (16–18). When considering IgG4-RD, keep in mind that storiform fibrosis and/or a high ratio of IgG4 plasma cells are distinctive for this disease, but are in no way specific. Other diseases that can mimic IgG4-RD have been reported, varying from inflammatory conditions (e.g., such as granulomatosis with poyangitis/Wegener's disease, Sjögren's disease and Castleman's disease) to malignancies (e.g., pancreatic adenocarcinoma and some lymphomas) (19–22). Therefore, a definitive diagnosis of IgG4-RD should always be made in collaboration with clinicians, and additional laboratory and radiological findings can support the diagnosis. If IgG4-RD is suspected in a specimen, but it does not fulfill the criteria mentioned in Table 1, additional evidence such as involvement of other organs or serum IgG4 <135 mg/dL are helpful to confirm the diagnosis. IgG4 serum levels in IgG4-RD patients are highly variable, but a mean value of 769 mg/dL was found in a systematic review including 349 patients. However, a significant proportion of patients with confirmed IgG4-RD have normal serum IgG4 levels (23). Furthermore, one should bear in mind that elevated IgG4 serum levels are also found in other disorders, such as granulomatosis with polyangiitis (Wegener's disease) and sarcoidosis (3).

**Table 1:** Criteria and findings in IgG4-RD.

**Major criteria (at least 2 out of 3)**	**Immunohistochemical findings**	**Supporting pathological findings**	**Supporting clinical findings**
Dense lympho-plasmacytic infiltrate	Relative count; IgG4 PC/IgG PC ratio ≥ 0.4	Presence of eosinophils	Multiple organ involvement
Storiform fibrosis	Absolute count; Amount of IgG4 PC/HPF^1^	Absence of neutrophils^2^	Serum IgG4 levels> 135 mg/dL
Obliterative phlebitis		Absence of necrosis	
		Absence of granulomas	
		Absence of multinucleated giant cells	

*Ad. 1. Varies according to organ localization (18)*.

*Ad. 2. With the exception of IgG4-RD in the lung, where intra-alveolar neutrophils can be present*.

*PC, plasma cells; HPF, high power field*.

Glucocorticosteroids are the first line of therapy for IgG4-RD, inducing a rapid response in the majority of patients. Unfortunately, sustained remission is infrequent, with a relapse rate reported between 40 and 77% of patients (2, 6). In case of recurrent or refractory disease, glucocorticoid-sparing agents such as azathioprine and methotrexate have been shown to be an effective treatment in individual cases (3). Rituximab is a very promising, corticosteroid sparing drug, yet it's efficacy has so far only been described in case reports, case series and an open label trial, and longterm maintenance therapy seems necessary for maintaining lasting disease remissions (24, 25).

A randomized controlled trial recently finished enrolling patients (https://clinicaltrials.gov/ct2/show/NCT01584388).

In conclusion, this case illustrates the occurrence of IgG4-RD presenting with sclerotic, FDG-avid bone marrow lesions and evolvement of an epidural mass in the thoracic spine without any other organ involvement, but with systemic symptoms. Laboratory findings and histopathological findings were consistent with IgG4-RD as described previously in other organs. The non-typical presentation with lesions in the spine and bone marrow resulted in diagnostic and therapeutic delay, yet disease symptoms resolved on initiation of treatment.

## Data Availability

All datasets analyzed for this study are included in the manuscript and/or the supplementary files.

## Ethics Statement

This article presents a case report. Patient gave written informed consent to publish the data. No medical ethic committee was involved in this publication.

## Author Contributions

MdB and RL suggested the diagnosis on histopathology. The diagnosis was confirmed clinically by DvdE and HL who managed the patient. DvdE and CS carried out a literature search, collected data about the case, and drafted the manuscript. TL generated Figure 1. CS and RL generated Figure 2. RL, TL, and HL reviewed and edited the manuscript. All authors approved the final version to be published and agrree to be accountable for all aspects of the work in ensuring that questions related to the accuracy or integrity of any part of the work are appropriately investigated and resolved.

### Conflict of Interest Statement

The authors declare that the research was conducted in the absence of any commercial or financial relationships that could be construed as a potential conflict of interest.
